# Not All That Shines on a PET Scan Is Cancer: A Silicone-Induced Granuloma Masquerading as Malignancy

**DOI:** 10.3390/clinpract11010003

**Published:** 2020-12-29

**Authors:** Krishna Vedala, Philip T. Sobash, Deborah Johnson, Krishna Kakkera

**Affiliations:** 1 Department of Internal Medicine, White River Health System, 1710 Harrison St, Batesville, AR 72501, USA; kvedala@wrmc.com; 2 Department of Pathology, White River Health System, 1710 Harrison St, Batesville, AR 72501, USA; djohnson@wrmc.com; 3 Department of Pulmonology, White River Health System, 1710 Harrison St, Batesville, AR 72501, USA; kkakkera@wrmc.com

**Keywords:** siloconomas, radiology, malignancy, pet imaging, false positives

## Abstract

PET/CT scans are frequently used in the initial workup of suspicious lesions but not all that lights up on a PET is cancerous. We wish to discuss a case of silicone-induced granuloma mimicking malignancy and the role of other imaging modalities for further workup.

## 1. Introduction

In a newly suspected diagnosis of lung cancer, ultrasound, X-ray, computed tomography (CT), magnetic resonance imaging (MRI), and positron emission tomography (PET) all play key roles in treatment planning. PET imaging in particular is useful for staging and practical information regarding the location of a suspected cancer. This, combined with CT can add further anatomical identification for further planning in treatment management [1]. PET scans utilize fludeoxyglucose in order to help locate areas of potential malignancy [2]. Not all that lights up on a PET scan can or should be considered malignancy, and clinical context can become even more pertinent. Inflammatory cells, sarcoidosis, and other thoracic etiologies such as silicone-induced granuloma, can cause PET scans to light up, giving false positives [3,4,5]. This false positivity when searching for malignancy can cloud diagnosis and can delay treatment, especially when risk factors indicate a likelihood of cancer.

## 2. Case Presentation

**Figure 1:**
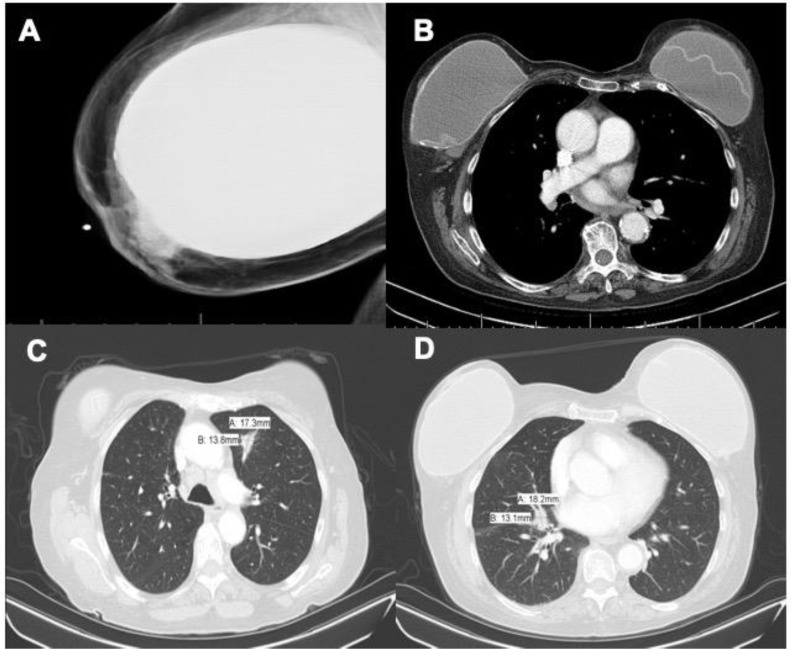
(**A**) Mammogram demonstrating extracapsular rupture of the right breast, (**B**) mediastinal window chest CT demonstrating Linguine Sign of the left breast implant, (**C**) chest CT showing an 18 mm × 14 mm nodule in the right middle lobe causing concern for malignancy, and (**D**) the same image showing a 17 mm × 14 mm spiculated left lung nodule with concern for breast involvement.

**Figure 2:**
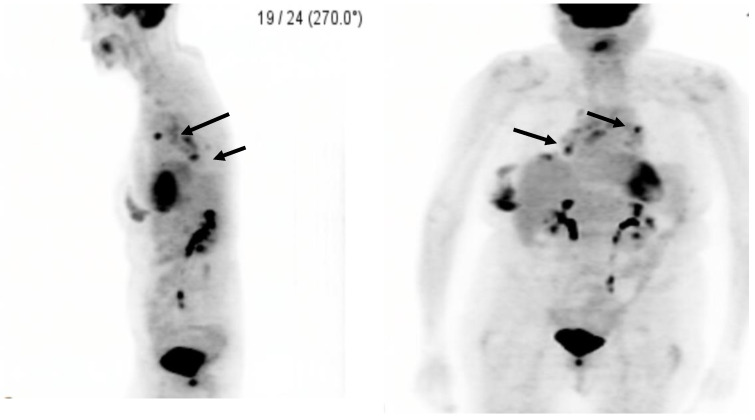
PET/CT demonstrating increased uptake with multiple mediastinal lymph nodes with a nodule in the right middle lobe and a spiculated left lung nodule with concern for breast involvement: the black arrows point to areas of suspicion.

Our patient is a 75-year-old female with history of hypertension, hyperlipidemia, depression, chronic pain syndrome, chronic obstructive pulmonary disease (COPD), former smoking for 58 pack-years, and breast implantation (cosmetic) over 20 years go. Her initial presenting symptoms were dyspnea with shortness of breath on exertion. Due to her extensive smoking history, she received a screening chest CT which revealed a spiculated left lung nodule with mediastinal adenopathy and Linguine Sign causing concern for intracapsular silicone implant rupture (Figure 1B–D). Due to concern for lung cancer, a subsequent PET scan identified significant uptake in the lesion seen on the CT with the left upper lobe with MAX SUV 6.3 and a smaller focus of opacity was noted in the right upper lobe with MAX SUV 2.7 (Figure 2). Along with this, there was hypermetabolic activity in the right breast also appreciated. At this point, there was concern for breast or, more likely, lung cancer.

She was referred for a breast ultrasound, which showed extracapsular silicone implant rupture. A mammogram of the right breast was performed, which was also negative for malignancy although it did confirm extracapsular implant rupture and extruded silicone (Figure 1A). She was referred to pulmonology for further workup of the lung nodules with endobronchial ultrasound (EBUS). Biopsy of multiple mediastinal nodes showed histiocytosis with possible granuloma formation. A CT-guided biopsy of the left lung nodule demonstrated more non-caseating epithelioid granulomas. The patient was prescribed a short course of Prednisone with instructions to follow-up in 6 months with surveillance imaging.

## 3. Discussion

**Figure 3:**
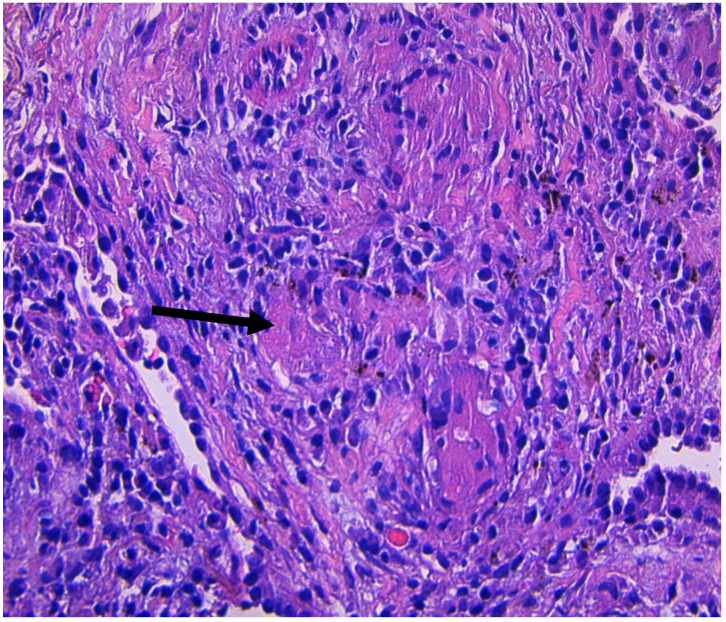
Non-caseating granuloma from a CT-guided biopsy of the left lung nodule showing an asteroid body (black arrow), a typical finding in granulomas.

Silicone breast implants were utilized beginning in the 1960s [6]. The first reported cases in the literature of silicone granulomas were reported by Winer et al. in 1964 [7]. Granulomas from ruptured implants have been reported previously and have been shown to have a wide variety of acute and chronic symptoms [8]. There is still much speculation around the pathophysiology of silicon-induced granuloma of breast implant (SIGBIC). It occurs in approximately 27% of individuals who have a silicone breast implant [9]. These silicone particles are believed to cause an autoimmune response increasing T-cell activation. If there is migration of the particles into the lymph nodes, this can result in an adjuvant effect, leading to localized inflammation and granuloma formation [10,11]. Histology from SIGBIC consists of histiocytes, granulomatous infiltrations, and a mix of lymphocytes such as T-cells and B-cells [10]. This can be seen in Figure 3 with the mix of lymphocytes and granuloma formation along with asteroid bodies specific for granuloma. What makes certain cases harder to diagnose is the presence of spiculation seen on CTs. While not specific for breast cancer, it is clinically correlated with cancer and makes the diagnosis highly suspicious [12,13].

Ali et al. presented the case of a 66-year-old female also with history of breast augmentation surgery found with lethargy, weight loss, and anorexia. CT imaging showed 2 suspicious pulmonary nodules, while a mammogram revealed a spiculated 4.1-cm mass. Malignancy was ruled out after Ultrasound (US-)guided biopsy showed silicone granulomas without any microcalcifications or breast tissue. [14] Grubstein et al. described two cases of siliconomas manifesting as multiple pulmonary nodules. In both cases, the masses showed significantly elevated Flourodeoxyglucose (FDG) uptake on the PET scan, but malignancy was eventually ruled out by biopsy [15]. Neither of these cases utilized a PET scan during workup. These cases highlight the variance of what an implant-induced silicone granuloma can present and their mimicry of cancer.

What makes our case uniquely challenging is that the patient was at high risk for lung malignancy because of the extensive smoking history coupled with her age. This case illustrates that findings on PET/CT do not necessarily indicate malignancy, especially in patients with a history of ruptured silicone breast implants. The literature supports that PET scans are not the optimal imaging modality if there is suspicion of silicone granulomas due to their high chance of false positives for malignancy. MRI is more useful in imaging for silicone-induced granuloma formation compared to mammography and US, even when there is no capsule rupture [16]. In conclusion, we recommend that, when working up siliconoma from ruptured breast implants in the context of possible malignancy, performing an MRI first would be more prudent even if a PET/CT is later warranted.

## Data Availability

The data presented in this study are available on request from the corresponding author. The data are not publicly available in order to protect patient information and confidentiality.
